# Predictors of neuropsychiatric manifestations in pediatric patients with lupus

**DOI:** 10.1371/journal.pone.0325915

**Published:** 2025-06-10

**Authors:** Wei Jiang, Xin Peng, Liqun Dong, Ling Wu, Yazhen Di, Li Lin

**Affiliations:** 1 Department of Pediatric Rheumatology and Immunology, Women’s and Children’s Hospital of Ningbo University, Ningbo City, Zhejiang Province, China; 2 Division of Pediatric Pulmonology and Immunology, West China Second University Hospital, Sichuan University, Chengdu, Sichuan, China; 3 Key Laboratory of Birth Defects and Related Diseases of Women and Children, Sichuan University, Ministry of Education, Chengdu, Sichuan, China; 4 NHC Key Laboratory of Chronobiology, Sichuan University, Chengdu, Sichuan, China; Transilvania University of Brasov: Universitatea Transilvania din Brasov, ROMANIA

## Abstract

Early detection of neuropsychiatric systemic lupus erythematosus (NPSLE) in children remains challenging in clinical settings. This study aims to describe the autoantibodies, organ disorders, the SLEDAI-2K score, and complement levels at the time of diagnosis in systemic lupus erythematosus (SLE) as well as investigate the predictors of NPSLE. We reviewed medical records of hospitalized children with SLE (< 18 years old) and extracted information on clinical features, serum autoantibodies, and laboratory test results. Multivariable logistic regression was used to establish the predictors of NPSLE and SLE without neuropsychiatric manifestations. Results revealed that 22.8% of children with NPSLE had higher ANA levels and SLEDAI-2K scores, lower C4 levels, and greater AMA-M2, β2GPI Abs, Anti-Rib-P Ab, ANCA, and LAC positivity at SLE diagnosis. The predictors of NPSLE were β2GPI-Abs (OR = 4.603), anti-Rib-P Ab (OR = 4.153), and SLEDAI-2K score (OR = 1.215). In summary, the findings show that the SLEDAI-2K score, β2GPI-Abs, and anti-Rib-P Ab are predictors of NPSLE.

## Introduction

Systemic lupus erythematosus (SLE) is a chronic autoimmune disease targeting any organ system, causing significant morbidity and mortality. Childhood-onset SLE is a lifelong autoimmune disease with a more aggressive course than adult-onset SLE, with increased disease activity at presentation and over time, resulting in higher morbidity and mortality rates than adult-onset SLE [1]. Patients with SLE, who exhibit one or more neuropsychiatric symptoms, have a subtype of SLE known as neuropsychiatric systemic lupus erythematosus (NPSLE). Between 22% and 95% of children with SLE develop NPSLE, which appears more severe in children and mostly affects the central nervous system (CNS), with peripheral and autonomic nervous system involvement being rare. NPSLE may be linked to higher rates of permanent organ damage among children than in adults [2–4].

NPSLE is difficult to diagnose and treat in daily clinical practice, causing severe, irreversible neuropsychiatric (NP) damage [5]. Furthermore, the lack of specific biomarkers for diagnostic or prognostic purposes complicates NPSLE management, and detecting its early NP symptoms requires a high level of clinical suspicion [6]. To address these issues, several interventions have been proposed, including biomarkers, neuroimaging, and diagnostic algorithms [7]. However, no precise procedure for managing NPSLE has been established.

Therefore, clinicians must recognize the predictors of NPSLE in children. Nonetheless, evidence on the risk factors for developing NPSLE in children is scarce, which is critical for diagnosing this disease and predicting its prognosis. In this regard, this study describes the characteristics and risk factors of pediatric NPSLE to provide a reference for predicting its occurrence and early detection, thereby promoting early intervention to improve its prognosis.

## Materials and methods

### Patients

This is a retrospective cross-sectional multicenter study. The medical records of all patients with SLE admitted to the West China Second University Hospital, Sichuan University, Chengdu, China, and the Women and Children’s Hospital of Ningbo University, Ningbo, China, were reviewed between January 2022 and December 2023.

SLE was diagnosed by a pediatric rheumatologist based on the American College of Rheumatology (ACR) 1997 criteria [8]. Patients were diagnosed with NPSLE according to the ACR nomenclature and case definitions published in 1999 [9]. A pediatric rheumatologist and a pediatric neurologist diagnosed and classified NPSLE. Many patients underwent neurological examinations, including brain magnetic resonance imaging, computed tomographic scanning of the head, electroencephalograms, and cerebrospinal fluid examination. We excluded the effects of drugs, CNS infection, tumors, and metabolic derangements before diagnosing NPSLE. Based on the ACR Nomenclature of 19 NPSLE syndromes, these NP syndromes can be divided into 12 central nervous system (CNS) and 7 peripheral nervous system (PNS) [9]. The 12 CNS included Seizure disorder, Aseptic meningitis, Demyelinating syndromes, Myelopathy, Headache, Cerebrovascular disease, Movement disorders, Anxiety disorders, Psychosis, Acute confusional state, Cognitive dysfunction, and Mood disorders. The 7 PNS included Autonomic disorders, Myasthenia gravis, Polyneuropathy, Cranial neuropathy, Guillian Barre syndrome, Mononeuropathy, and Plexopathy.

Cognitive dysfunction was evaluated based on the test combination proposed by the ACR in 1999. It was defined as a decline in function in one or more of the following domains: simple or complex attention, memory (such as learning and recall), visual-spatial processing, language (such as language fluency), reasoning and problem-solving, psychomotor speed, and executive functions (such as planning, organizing, and sequencing) [9]. The cognitive function was quantitatively evaluated using the ACR neuropsychological test combination and the Cognitive Symptom Inventory (CSI) [10]. Anxiety and depression were the two aspects most closely related to mood disorders [11]. Herein, the assessment of mood disorders was based on the Hospital Anxiety and Depression Scale (HADS) and the 1999 ACR criteria [9,12]. Symptoms of autonomic disorder include dizziness, palpitation, abnormal sweating, abnormal blood pressure, constipation, etc. We recorded the manifestations of autonomic nerve function based on the clinical manifestations and Composite Autonomic Symptom Score (COMPASS)-31 questionnaire [13] and confirmed the diagnosis of autonomic disorder. According to the 1999 ACR NPSLE nomenclature [9], movement disorders included chorea, athetosis, hemibalismus, cerebellar ataxia, Parkinson-like, etc. Tension headaches and migraine accounted for the majority of the headache symptoms. Seizure disorders included generalized seizures, focal seizures, etc. The acute confusional state included confusion, delirium, etc. Cerebrovascular disease included paralysis of limbs, aphasia, sensory disorders, etc. The neuropsychiatric symptoms of NPSLE, including headache, seizure disorders, acute confusional state, movement disorder, and cerebrovascular disease, were diagnosed based on the International Classification of Diseases 10^th^ Edition (ICD-10) [14] and the 1999 ACR NPSLE nomenclature [9].

The disease activity score was evaluated using the SLE disease activity index-2000 (SLEDAI-2K) [15] before treatment. Clinical manifestations and laboratory data of the patients were extracted from their medical records.

### Selection of autoantibodies

The increased level of the antinuclear antibody (ANA), anti-double-strand DNA antibody (anti-dsDNA Ab), and anti-histone antibody (anti-HI Ab) correlated with disease activity [16]. Anti-ribosomal-P antibody (anti-Rib-P Ab) was specifically associated with psychosis in NPSLE [17]. Anti-cardiolipin antibodies (aCL Abs) and lupus anticoagulant (LAC) were associated with cognitive impairments in NPSLE, whereas Anti-beta2-glycoprotein I antibodies (β2GPI Abs) were associated with various neuropsychiatric symptoms [18]. High levels of Anti-Smith antibody (anti-Sm Ab) correlated with acute confusional state and disruption of the blood-brain barrier (BBB) [16]. Meulen PM et al. found anti-centromere protein antibody (anti-CENP Ab) at significantly different levels in NPSLE [19]. Anti-nucleosome antibodies (anti-NU Ab) were more positive among NPSLE patients than in non-NPSLE [20]. Anti-proliferating cell nuclear antigen antibody (anti-PCNA Ab) was found using a human proteome microarray-based approach, and validated as a biomarker in CSF of SLE patients [21]. The presence of anti-Sjogren’s syndrome A antibody (anti-SSA Ab) is linked to NPSLE, whereas the presence of anti-Sjogren’s syndrome B antibody (anti-SSB Ab) was not associated with NPSLE [16]. Anti-U1 small nuclear ribonucleoprotein antibody (anti-U1RNP Ab) showed no correlation with neuropsychiatric manifestations [16]. We found a high prevalence of anti-mitochondrial antibody-M2 (AMA-M2) in patients with subacute cutaneous lupus erythematosus [22]. The existence of anti-neutrophil cytoplasmic antibodies (ANCA) is linked to a higher level of disease activity in SLEDAI-2K [23]. Anti-scleroderma-70 antibody (anti-Scl70 Ab) is a scleroderma-specific autoantibody associated with an increased risk of interstitial lung disease (ILD) [24], whereas anti-polymyositis-sclerosis antibody (anti-PM Scl Ab) is an autoantibody associated with Systemic Sclerosis (SSc) [25].

### Data collection

Pediatric patient data were collected from the electronic medical record system. Data from West China Second University Hospital, Sichuan University, were accessed and obtained between July 1, 2024, and July 31, 2024. Similarly, data from the Women and Children’s Hospital of Ningbo University were also accessed and obtained during the same period. All data were collected and organized based on the requirements of the research protocol. This was after obtaining the corresponding ethical approval and patient informed consent. Relevant patient data at the time of SLE diagnosis, including demographic information (age, sex, and the duration between disease onset and diagnosis), clinical manifestations (fever, rash, organic disorder, and SLEDAI-2K), and laboratory test results (complement, autoantibodies, cerebrospinal fluid, and imaging test results) were extracted and collated in a standardized, anonymized data collection spreadsheet. We also obtained clinical findings in patients with NPSLE, such as CNS manifestations and peripheral nervous system manifestations based on the 1999 ACR [9].

### Data analysis

All analyses were carried out using the SPSS software (version 26.0; IBM Corporation, Armonk, NY, USA). A Kolmogorov-Smirnov test was used to test for the normality of continuous numerical variables, and normally distributed data were expressed as mean and standard deviation (x ± s). For non-normally distributed continuous numerical variables, the data were expressed as the median with the upper and lower quartiles (M [P25, P75]), and the rank sum test was used for comparison. Count data were described as n (%), and a Chi-square test was used to compare differences between groups. The Pearson Chi-square test was used as the nonparametric test where the sample size was less than 40 and the theoretical frequency was less than 5. A Fisher’s exact probability test was performed if the sample size was < 40 or the theoretical frequency was < 5. A value of 0.05 was used as the test level, and a P < 0.05 was considered statistically significant. Logistic regression analyses (univariate and multivariate analyses) were performed to determine the risk factors for NPSLE.

### Ethics approval and consent to participate

Ethical clearance was obtained from both the Research and Institutional Review Board of West China Second University Hospital of Sichuan University (approval NO. EC2024-156) and the Research and Institutional Review Board of Women and Children’s Hospital of Ningbo University (approval NO. EC2024-091). Since it was a retrospective study, information about the participants remained confidential, and a strict code of ethics was enforced. The need for informed consent to participate was waived by the Institutional Review Board in both two centers (West China Second University Hospital of Sichuan University and Women and Children’s Hospital of Ningbo University). This study was conducted in adherence to the principles of the Declaration of Helsinki. The research protocol conformed with the standards currently applied in China.

## Results

### Patient characteristics

A total of 101 pediatric patients newly diagnosed with SLE were identified during the study period. Table 1 shows their demographics (Table 1). In total, 85 were females and 16 were males, with a sex ratio of 84:16. The mean age at the onset of SLE was 10.76 ± 2.35 years, whereas the mean time from onset to diagnosis was 61.74 ± 97.51 days. A total of 13 (12.9%), 17 (16.8%), and 71 (70.3%) patients had a SLEDAI-2K score of ≤ 6, 7–12, and > 12, respectively. The ANA level was 1:100, 1:320, 1:1000, and 1:3200 in 8 (7.9%), 16 (15.8%), 42 (41.6%), and 35 (34.7%) patients, respectively. We also examined the complement levels and found that the mean C3 level of 0.49 ± 0.38, whereas the mean C4 level was 0.08 ± 0.07. The proportion of patients positive for anti-dsDNA Ab, anti-Sm Ab, anti-CENP Ab, anti-PCNA Ab, anti-NU Ab, anti-HI Ab, anti-Rib-P Ab, AMA-M2, anti-U1RNP Ab, anti-SSA Ab, anti-SSB Ab, anti-Scl70 Ab, anti-PM Scl Ab, ANCA, LAC, aCL Abs, and β2GPI Abs were 61.4%, 32.7%, 3.0%, 2.0%, 59.4%, 51.5%, 29.7%, 28.7%, 41.6%, 38.6%, 20.8%, 4.0%, 12.9%, 46.5%, 32.7%, 21.8%, and 23.8%, respectively. We also assessed the involvement of organs and systems in children with lupus at the time of diagnosis. As a result, 57.4% of children had a skin rash, 52.5% had a fever, and 4.0% had MAS. Among children with lupus, 73.3% had a renal disorder, 71.3% had a hematologic disorder, 14.9% had arthritis, 19.8% had a pulmonary disorder, 12.9% had a cardiac disorder, 30.7% had a digestive disorder, 35.6% had hypothyroidism, and 22.8% had a neurologic disorder (S1 Table).

**Table 1:** Demographic, clinical, and ancillary examination characteristics of all studied children with SLE.

Characteristics	N = 101
Time from onset to diagnosis (days) mean (± SD)	61.74 ± 97.51
Age (years) mean (± SD)	10.76 ± 2.35
Sex *n* (%)	
Male	16 (15.8)
Female	85 (84.2)
SLEDAI-2K, mean (± SD)	16.53 ± 8.09
SLEDAI-2K *n* (%) (≤ 6)	13 (12.9)
SLEDAI-2K *n* (%) (7–12)	17 (16.8)
SLEDAI-2K *n* (%) (> 12)	71 (70.3)
C3, mg/dl, mean (± SD)	0.49 ± 0.38
C4, mg/dl, mean (± SD)	0.08 ± 0.07
ANA positive, *n* (%)	
1:100	8 (7.9)
1:320	16 (15.8)
1:1000	42 (41.6)
1:3200	35 (34.7)
Anti-dsDNA Ab *n* (%)	62 (61.4)
Anti-Sm Ab *n* (%)	33 (32.7)
Anti-CENP Ab *n* (%)	3 (3.0)
Anti-PCNA Ab *n* (%)	2 (2.0)
Anti-NU Ab *n* (%)	60 (59.4)
Anti-HI Ab *n* (%)	52 (51.5)
Anti-Rib-P Ab *n* (%)	30 (29.7)
AMA-M2 *n* (%)	29 (28.7)
Anti-U1RNP Ab *n* (%)	42 (41.6)
Anti-SSA Ab *n* (%)	39 (38.6)
Anti-SSB Ab n (%)	21 (20.8)
Anti-Scl70 Ab *n* (%)	4 (4.0)
Anti-PM Scl Ab *n* (%)	13 (12.9)
ANCA *n* (%)	47 (46.5)
LAC *n* (%)	33 (32.7)
aCL Abs *n* (%)	22 (21.8)
β2GPI Abs *n* (%)	24 (23.8)
Rash *n* (%)	58 (57.4)
Fever *n* (%)	53 (52.5)
MAS *n* (%)	4 (4.0)
Renal disorder *n* (%)	74 (73.3)
Hematologic disorder *n* (%)	72 (71.3)
Arthritis *n* (%)	15 (14.9)
Pulmonary disorder *n* (%)	20 (19.8)
Cardiac disorder *n* (%)	13 (12.9)
Digestive disorder *n* (%)	31 (30.7)
Hypothyroidism *n* (%)	36 (35.6)
Neurologic disorder *n* (%)	23 (22.8)
NP manifestations *n* (%)	23 (22.8)
NP characteristics	
Headache	8 (34.8)
Seizure disorders	1 (4.3)
Cognitive dysfunction	2 (8.7)
Acute confusional state	1 (4.3)
Movement disorder	3 (13.0)
Mood disorder	6 (26.1)
Autonomic disorder	6 (26.1)
Cerebrovascular disease	4 (17.4)
NP auxiliary inspection	
Abnormal brain MRI	22 (95.6)
Abnormal electroencephalogram	4 (17.4)

Unless otherwise indicated, values are expressed as numbers (percentages). All antibodies show the number of participants that were positive. *ANA* antinuclear antibody, *SLEDAI-2K* SLE disease activity index-2000, *anti-dsDNA Ab* anti-double-stranded DNA antibody, *anti-Sm Ab* anti-Smith antibody, *anti-CENP Ab* anti-centromere protein antibody, *anti-PCNA Ab* anti-proliferating cell nuclear antigen antibody, *anti-NU Ab* anti-nucleosome antibody, *anti-HI Ab* anti-histone antibody, *anti-Rib-P Ab* anti-ribosomal-P antibody, *AMA-M2* anti-mitochondrial antibody-M2, *anti-U1RNP Ab* anti-U1 small nuclear ribonucleoprotein antibody, *anti-SSA Ab* anti-Sjogren’s syndrome A antibody, *anti-SSB Ab* anti-Sjogren’s syndrome B antibody, *anti-Scl70 Ab* anti-scleroderma-70 antibody, *anti-PM Scl Ab* anti-polymyositis-sclerosis antibodies, *ANCA* anti-neutrophil cytoplasmic antibody, *LAC* lupus anticoagulant, *aCL Abs* anti-cardiolipin antibodies, *β2GPI Abs* anti-beta2-glycoprotein I antibodies, *MAS* macrophage activation syndrome, *MRI* magnetic resonance imaging, *NP* neuropsychiatric disorders.

### Clinical features of NPSLE

Among the 101 children with SLE, 23 had NPSLE. Most children with NPSLE had a headache (34.8%), a mood disorder (26.1%), or an autonomic disorder (26.1%), and several had cerebrovascular disease (17.4%), movement disorders (13.0%), cognitive dysfunction (8.7%), seizure disorders (4.3%), and an acute confusional state (4.3%). Radiological examination revealed that 95.6% of them had abnormal brain magnetic resonance imaging results, and 17.4% had an abnormal electroencephalogram (Table 1). Among the 23 NPSLE patients, 22 of them (95.6%) showed abnormal results in the brain magnetic resonance imaging. These included 15 presenting with parenchyma abnormal signal, 3 with atrophy, and 4 exhibiting both parenchyma abnormal signal and atrophy (S2 Table).

NPSLE occurred in 23 (22.8%) children with SLE, aged 11.1 ± 2.02 years at diagnosis. Among those with NPSLE, 91.3% were females, whereas female patients accounted for 82.1% of those without NPSLE (P = 0.352) (Table 2).

**Table 2:** Clinical manifestations in patients with systemic lupus erythematosus (SLE) with or without neuropsychiatric (NP) manifestations.

Variables	Without NP manifestations (n = 78)	With NP manifestations (n = 23)	P-value
Time from onset to diagnosis, days mean (± SD)	57.42 ± 91.42	76.39 ± 116.94	0.479
Age, years, mean (± SD)	10.65 ± 2.44	11.1 ± 2.02	0.376
Sex, *n* (%)			0.352
Male	14 (17.9)	2 (8.7)	
Female	64 (82.1)	21 (91.3)	
SLEDAI-2K score, mean (± SD)	14.46 ± 7.26	23.57 ± 6.77	< 0.001*
^a^SLEDAI-2K score *n* (%)			0.007*
≤ 6	13 (16.7)	0 (0)	
7–12	16 (20.5)	1 (4.3)	
> 12	49 (62.8)	22 (95.7)	
C3, mg/dl, mean (± SD)	0.51 ± 0.35	0.42 ± 0.49	0.444
C4, mg/dl, mean (± SD)	0.08 ± 0.08	0.05 ± 0.05	0.041*
^b^ANA positive *n* (%)			0.025*
1:100	7 (9)	1 (4.3)	
1:320	15 (19.2)	1 (4.3)	
1:1000	35 (44.9)	7 (30.4)	
1:3200	21 (26.9)	14 (60.9)	
Anti-dsDNA Ab *n* (%)	44 (56.4)	18 (78.3)	0.099
Anti-Sm Ab *n* (%)	25 (32.1)	8 (34.8)	1
Anti-CENP Ab *n* (%)	1 (1.3)	2 (8.7)	0.129
Anti-PCNA Ab *n* (%)	1 (1.3)	1 (4.3)	0.405
Anti-NU Ab *n* (%)	44 (56.4)	16 (69.6)	0.375
Anti-HI Ab *n* (%)	37 (47.4)	15 (65.2)	0.207
Anti-Rib-P Ab *n* (%)	17 (21.8)	13 (56.5)	0.003*
AMA-M2 *n* (%)	18 (23.1)	11 (47.8)	0.041*
Anti-U1RNP Ab *n* (%)	31 (39.7)	11 (47.8)	0.652
Anti-SSA Ab *n* (%)	31 (39.7)	8 (34.8)	0.853
Anti-SSB Ab *n* (%)	13 (16.7)	8 (34.8)	0.08
Anti-Scl70 Ab *n* (%)	3 (3.8)	1 (4.3)	1
Anti-PM Scl Ab *n* (%)	7 (9)	6 (26.1)	0.069
ANCA *n* (%)	31 (39.7)	16 (69.6)	0.022*
LAC *n* (%)	19 (24.4)	14 (60.9)	0.002*
aCL Abs *n* (%)	14 (17.9)	8 (34.8)	0.152
β2GPI Abs *n* (%)	14 (17.9)	10 (43.5)	0.025*
Rash *n* (%)	43 (55.1)	15 (65.2)	0.535
Fever *n* (%)	35 (44.9)	18 (78.3)	0.01*
MAS *n* (%)	0 (0)	4 (17.4)	0.002*
Renal disorder, *n* (%)	56 (71.8)	18 (78.3)	0.728
Hematologic disorder *n* (%)	52 (66.7)	20 (87)	0.104
Arthritis *n* (%)	9 (11.5)	6 (26.1)	0.101
Pulmonary disorder *n* (%)	13 (16.7)	7 (30.4)	0.231
Cardiac disorder *n* (%)	9 (11.5)	4 (17.4)	0.486
Digestive disorder *n* (%)	18 (23.1)	13 (56.5)	0.005*
Hypothyroidism *n* (%)	23 (29.5)	13 (56.5)	0.033*

*A P-value is significant if it is < 0.05. Values are presented as numbers (percentages) unless otherwise stated. The count data were described by n (%), and the chi-square test was used to compare variables between the groups. The sample size was > 40 and the theoretical frequency was > 5, and the Pearson chi-square test was used for nonparametric tests; for cases where the sample size was < 40 or the theoretical frequency was < 5, the Fisher exact probability method was used.

*SLEDAI-2K* SLE disease activity index-2000, *ANA* antinuclear antibody, *anti-dsDNA Ab* anti-double-stranded DNA antibody, *anti-Sm Ab* anti-Smith antibody, *anti-CENP Ab* anti-centromere protein antibody, *anti-PCNA Ab* anti-proliferating cell nuclear antigen antibody, *anti-NU Ab* anti-nucleosome antibody, *anti-HI Ab* anti-histone antibody, *anti-Rib-P Ab* anti-ribosomal-P antibody, *AMA-M2* anti-mitochondrial antibody-M2, *anti-U1RNP Ab* anti-U1 small nuclear ribonucleoprotein antibody, *anti-SSA Ab* anti-Sjogren’s syndrome A antibody, *anti-SSB Ab* anti-Sjogren’s syndrome B antibody, *anti-Scl70 Ab* anti-scleroderma70 antibody, *anti-PM Scl Ab* anti-polymyositis-sclerosis antibodies, *ANCA* anti-neutrophil cytoplasmic antibody, *LAC* lupus anticoagulant, *aCL Abs* anti-cardiolipin antibodies, *β2GPI Abs* anti-beta2-glycoprotein I antibodies, *MAS* macrophage activation syndrome

^a^A SLEDAI-2K score of ≤ 6 is classified as mild disease activity, 7–12 as moderate disease activity, and > 12 as severe disease activity.

^b^Positive antinuclear antibodies with a titer of ≥ 1:100 on indirect immunofluorescence

When children with SLE alone were compared with those with NPSLE (Table 2), children with NPSLE were more likely to develop a fever (44.9% vs. 78.3%, P = 0.01), a higher SLEDAI-2K score (14.46 ± 7.26 vs. 23.57 ± 6.77, P < 0.001), higher ANA levels (P = 0.025), and lower C4 levels (0.08 ± 0.08 vs. 0.05 ± 0.05, P = 0.041) than those of children with SLE alone. Anti-Rib-P Ab, AMA-M2, and ANCA were more frequently positive in children with NPSLE than in children with SLE alone (21.8% vs. 56.5%, P = 0.003; 23.1% vs. 47.8%, P = 0.041; and 39.7% vs. 69.6%, P = 0.022; respectively). For antiphospholipid antibodies, the LAC and β2GPI Abs were more frequently positive in children with NPSLE than in those with SLE alone (24.4% vs. 60.9%, P = 0.002, and 17.9% vs. 43.5%, P = 0.025, respectively). Regarding organ and system involvement, children with NPSLE were prone to digestive disorders (23.1% vs. 56.5%, P = 0.005), MAS (0 vs. 17.4%, P = 0.002), and hypothyroidism (29.5% vs. 56.5%, P = 0.033) compared to those with SLE alone (Table 2).

### Risk factors for NPSLE

Further, we sought to identify relationships between clinical or laboratory data and neurologic involvement in SLE diagnosis. In univariate logistic regression analyses, the presence of anti-Rib-P Ab (OR = 4.665, 95% CI 1.743–12.481; P = 0.002), anti-SSA Ab (OR = 3.168, 95% CI 1.197–8.384; P = 0.02), AMA-M2 (OR = 3.056, 95% CI 1.155–8.085; P = 0.024), anti-PM Scl Ab (OR = 3.58, 95% CI 1.065–12.03; P = 0.039), ANCA (OR = 3.465, 95% CI 1.278–9.394; P = 0.015), β2GPI Ab (OR = 3.516, 95% CI 1.285–9.626; P = 0.014), aCL Ab (OR = 5.231, 95% CI 1.815–15.074; P = 0.002), or LAC (OR = 4.83, 95% CI 1.805–12.924; P = 0.002) was a significant risk factor for NPSLE. Children with positive ANA (OR = 1.005, 95% CI 1.001–1.111; P = 0.003) and a high SLEDAI-2K score (OR = 1.204, 95% CI 1.101–1.318; P < 0.001) were also more likely to develop NPSLE. Fever (OR = 4.423, 95% CI 1.492–13.111; P = 0.007), digestive disorders (OR = 4.333, 95% CI 1.629–11.526; P = 0.003), and hypothyroidism (OR = 3.109, 95% CI 1.193–8.097; P = 0.02) were also risk factors for NPSLE in children with lupus. In contrast, high serum complement (C3 and C4) levels were protective against NPSLE (C3: OR = 0.503, 95% CI 0.118–0.938; P = 0.035 and C4: OR = 0.024, 95% CI 0.009–0.982; P = 0.035) (Table 3).

**Table 3:** Univariate analyses of factors associated with NP dysfunction.

Variable	OR	95% CI OR		P-value
		Lower limit	Upper limit	
Time from onset to diagnosis, days	1.002	0.997	1.006	0.418
Age	1.091	0.884	1.346	0.419
Sex	2.297	0.482	10.946	0.297
SLEDAI-2K	1.204	1.101	1.318	< 0.001*
C3	0.503	0.118	0.938	0.035*
C4	0.024	0.009	0.982	0.035*
ANA	1.005	1.001	1.111	0.003*
Anti-dsDNA Ab	2.782	0.938	8.251	0.065
Anti-Sm Ab	1.131	0.424	3.016	0.806
Anti-CENP Ab	7.333	0.634	84.851	0.111
Anti-PCNA Ab	3.5	0.21	58.252	0.383
Anti-NU Ab	1.766	0.653	4.775	0.262
Anti-HI Ab	2.078	0.791	5.461	0.138
Anti-Rib-P Ab	4.665	1.743	12.481	0.002*
AMA-M2	3.056	1.155	8.085	0.024*
Anti-U1RNP Ab	1.39	0.545	3.542	0.49
Anti-SSA Ab	3.168	1.197	8.384	0.02*
Anti-SSB Ab	2.667	0.938	7.578	0.066
Anti-Scl70 Ab	1.136	0.113	11.477	0.914
Anti-PM Scl Ab	3.58	1.065	12.03	0.039*
ANCA	3.465	1.278	9.394	0.015*
LAC	4.83	1.805	12.924	0.002*
aCL Abs	5.231	1.815	15.074	0.002*
β2GPI Abs	3.516	1.285	9.626	0.014*
Rash	1.526	0.58	4.014	0.392
Fever	4.423	1.492	13.111	0.007*
Hematologic disorder	3.333	0.907	12.251	0.07
Renal disorder	1.414	0.468	4.278	0.539
MAS	6.543	0.001	95.245	0.999
Arthritis	2.706	0.847	8.643	0.093
Pulmonary disorder	2.187	0.751	6.373	0.151
Cardiac disorder	1.614	0.448	5.82	0.464
Digestive disorder	4.333	1.629	11.526	0.003*
Hypothyroidism	3.109	1.193	8.097	0.02*

*A P-value is significant if it is < 0.05. *OR* odds ratio, *CI* confidence interval, *SLEDAI-2K* SLE disease activity index-2000, *ANA* antinuclear antibody, *anti-dsDNA Ab* anti-double-stranded DNA antibody, *anti-Sm Ab* anti-Smith antibody, *anti-CENP Ab* anti-centromere protein antibody, *anti-PCNA Ab* anti-proliferating cell nuclear antigen antibody, *anti-NU Ab* anti-nucleosome antibody, *anti-HI Ab* anti-histone antibody, *anti-Rib-P Ab* anti-ribosomal-P antibody, *AMA-M2* anti-mitochondrial antibody-M2, *anti-U1RNP Ab* anti-U1 small nuclear ribonucleoprotein antibody, *anti-SSA Ab* anti-Sjogren’s syndrome A antibody, *anti-SSB Ab* anti-Sjogren’s syndrome B antibody, *anti-Scl70 Ab* anti-scleroderma 70 antibody, *anti-PM Scl Ab* anti-polymyositis-sclerosis antibodies, *ANCA* anti-neutrophil cytoplasmic antibody, *LAC* lupus anticoagulant, *aCL Abs* anti-cardiolipin antibodies, *β2GPI Abs* anti-beta2-glycoprotein I antibodies, *MAS* macrophage activation syndrome

**Fig 1:**
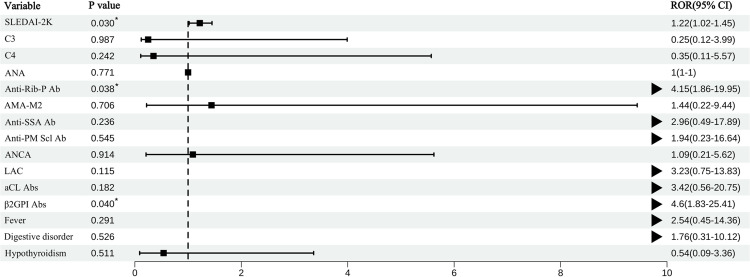
A forest map of multivariate analyses of factors associated with NP dysfunction. Note: When it was represented as an arrow, the multivariate analysis result of the variable was statistically significant (P < 0.05) and the 95% confidence interval of the OR value exceeded 10.

We carried out multivariate analyses for the factors significantly different from the univariate analysis. This revealed that a high SLEDAI-2K score (OR = 1.215, 95% CI 1.019–1.449; P = 0.03), the presence of β2GPI Ab (OR = 4.603, 95% CI 1.834–25.412; P = 0.04), and the presence of anti-Rib-P Ab (OR = 4.153, 95% CI 1.865–19.95; P = 0.038) were significantly associated with NPSLE (Table 4). The forest map was drawn for the results of the multi-factor logistics regression analysis, and we visualized the OR value and its 95% confidence interval (Fig 1).

**Table 4:** Multivariate analyses of factors associated with NP dysfunction.

Variable	OR	95% CI OR		P-value
		Lower limit	Upper limit	
SLEDAI-2K	1.215	1.019	1.449	0.03*
C3	0.254	0.115	3.99	0.987
C4	0.347	0.113	5.572	0.242
ANA	1.000	0.999	1.001	0.771
Anti-Rib-P Ab	4.153	1.865	19.95	0.038*
AMA-M2	1.436	0.218	9.436	0.706
Anti-SSA Ab	2.964	0.491	17.894	0.236
Anti-PM Scl Ab	1.942	0.227	16.645	0.545
ANCA	1.095	0.213	5.616	0.914
LAC	3.226	0.752	13.832	0.115
aCL Abs	3.418	0.563	20.75	0.182
β2GPI Abs	4.603	1.834	25.412	0.04*
Fever	2.542	0.45	14.355	0.291
Digestive disorder	1.761	0.306	10.125	0.526
Hypothyroidism	0.542	0.087	3.364	0.511

*A P-value is significant if it is < 0.05. *OR* odds ratio, *CI* confidence interval, *SLEDAI-2K* SLE disease activity index-2000, *ANA* antinuclear antibody, *anti-Rib-P Ab* anti-ribosomal-P antibody, *AMA-M2* anti-mitochondrial antibody-M2, *anti-SSA Ab* anti-Sjogren’s syndrome A antibody, *anti-PM Scl Ab* anti-polymyositis-sclerosis antibodies, *ANCA* anti-neutrophil cytoplasmic antibody, *LAC* lupus anticoagulant, *aCL Abs* anti-cardiolipin antibodies, *β2GPI Abs* anti-beta2-glycoprotein I antibodies

## Discussion

NP involvement is prevalent in pediatric SLE and is a leading cause of morbidity and mortality rates. In the present study, we analyzed the relationship between clinical variables and NPSLE and found contributing factors. The reported incidence of NP symptoms in SLE patients (adults and children) varies significantly between studies, ranging from 12% to 95% [26]. Prevalence estimates vary widely across studies. However, approximately 25% of pediatric patients with SLE appear to have NP involvement [4], consistent with our findings. We also noted that NPSLE occurred in 22.8% of children with SLE. Previous studies [27,28] found headache as one of the most frequent NP manifestations in SLE patients. Furthermore, a meta-analysis of studies assessing NPSLE prevalence by Unterman et al. [29] revealed that the most common manifestations of NPSLE included headache (28.3%), mood disorders (20.7%), and cognitive dysfunction (19.7%). Furthermore, headache (34.8%) was the most common NP manifestation, and mood disorders (26.1%) were the second most common NP manifestation in children with NPSLE; this was consistent with the findings of previous studies. Nonetheless, autonomic disorder (26.1%) was the second most common NP manifestation of NPSLE. This inconsistency with the results of previous studies is attributed to the fact that our study participants were all children who could not precisely describe their symptoms. Moreover, the manifestations of NPSLE may differ with age. In contrast to a previous study, 8.7% of children with NPSLE had calculation and cognitive dysfunction; this could be attributed to the fact that it was difficult to assess cognition and calculation skills in extremely young children.

The lupus disease activity score (SLEDAI-2K score) and cumulative organ damage were higher in the NPSLE group. These results are in line with the findings of previous studies [5,30–32], which documented more organ damage in the NPSLE group. This could be because of a relationship between SLE disease activity and concurrent diffuse as well as NP events attributable to SLE [33]. Fever is more common in younger patients with lupus, and NPSLE is more common in early-onset lupus [34,35]. Our findings revealed that children with NPSLE are more likely to have a fever than those with only SLE (P = 0.01). High ANA titers are commonly observed in patients with NPSLE [36]; the present study showed that children with NPSLE had a higher level of ANA than those with only SLE (P = 0.025). Shatri H et al. [37] discovered a negative correlation between anxiety and C3 or C4 levels in patients with SLE. Children with NPSLE had lower C3 and C4 levels (P = 0.444 and P = 0.041, respectively) than those with only SLE.

Becker Y et al. [38] revealed that in animal models, anti-mitochondrial RNA IgM titers strongly correlated with β2GPI-Abs (P < 0.0001). AMA-M2 and β2GPI-Abs were more frequently positive in the NPSLE group than in those with only SLE. Pan Y et al. [39] discovered that the appearance of perinuclear ANCA in SLE showed a high probability of lupus nephritis and a more severe condition. Moreover, patients with ANCA-positive lupus nephritis commonly have histological markers of severe disease [40]. ANCA was more positive in the NPSLE group than in the SLE alone group. This suggests that ANCA-positive patients have a higher disease activity and are more likely to have organ damage than ANCA-negative patients.

Anti-SSA antibodies are linked to subacute cutaneous lupus [41]. Novak GV et al. [42] reported that anti-Ro/SSA antibody is linked to childhood SLE with cutaneous and musculoskeletal involvements. On the other hand, Higuera-Ortiz V et al. [43] reported that anti-Ro/SSA antibodies may be involved in lupus-associated cardiac valve disease. Univariate logistic regression analysis in our study showed that anti-SSA Ab is a risk factor for NPSLE. Previous studies suggest that anti-PM-Scl antibodies are associated with an increased frequency of Raynaud’s phenomenon in patients with SLE [44]. Elsewhere, another study showed that dysphagia is associated with anti-PM-Scl antibodies in SLE [45]. Here, anti-PM Scl Ab was, however, a risk factor for NPSLE in univariate logistic regression analysis.

In addition, the anti-Rib-P Ab was more commonly positive in the NPSLE group than in the SLE alone group. This is consistent with the results from previous studies [46], reporting that being anti-Rib-P antibody positive is linked to the NP manifestations of SLE. Many researchers believe that anti-Rib-P antibodies are biomarkers of NPSLE [47–52]. However, Pradhan V et al. [53] found no statistically significant difference in the incidence of Rib-P antibodies between patients with NPSLE and those with SLE alone. These differences may be attributed to differences in race, region, and age.

After using a Chi-square test to compare the differences between SLE alone and NPSLE, univariate logistic regression was carried out to analyze the effect of single factors on NPSLE. Univariate logistic regression analyses showed that the presence of anti-Rib-P Ab, anti-SSA Ab, AMA-M2, anti-PM Scl Ab, ANCA, β2GPI Ab, aCL Ab, and LAC are significant risk factors for NPSLE. Risk factors for NPSLE included fever, positive ANA levels, and a high SLEDAI-2K score. High levels of C3 and C4 were the protective factors against NPSLE development. The results of the univariate logistic regression analyses were roughly consistent with those of the Chi-square test. Nonetheless, a logistic regression analysis better indicates the importance of the factor’s influence on the outcome variables [54].

According to a retrospective study in a Polish population by Pawlak-Buś K et al. [55], the SLEDAI‑2K score was significantly higher in patients with NPSLE than in those without NP symptoms attributable to SLE. Moreover, our study showed similar results, with the SLEDAI-2K score being higher among those with NPSLE than in those with SLE alone (P < 0.001); the SLEDAI-2K score was an independent risk factor for NPSLE. The NP domain scores of the EULAR/ACR criteria positively correlate with the SLEDAI-2K score [56]. Toledano P et al. [57] found that a high SLEDAI score is linked to white matter lesions and myelopathy in patients with NPSLE; this might explain the symptoms of patients with NPSLE.

The pivotal role of antiphospholipid (aPL) antibodies, comprising LAC, aCL Abs, and β2GPI-Abs, in NPSLE and their value in predicting progression in NP damage is popular [58–61]. Geng W et al. [62] recently found that β2GPI-Abs are more commonly observed in patients with mood disorders. In a previous meta-analysis by Ho RC et al. [63], β2GPI-Abs were significantly associated with mood disorders (OR = 6.27). Seth G et al. [64] reported that among patients with NPSLE, β2GPI-Abs is more commonly present in those with a stroke. Hawro T et al. [65] discovered that headaches and ischemic stroke are independently associated with β2GPI-Abs (OR = 5.6). All the above studies suggest that β2GPI-Abs may be a risk factor for NPSLE, corroborating our findings. In the multivariable analysis, β2GPI Ab was associated with a high risk of NPSLE. Early hypotheses and animal studies have shown that mood disorders may be attributed to neurotransmitter dysfunction [66], and recent research has shown that mood disorders may be caused by vascular dysfunction [67]. Moreover, recent studies have demonstrated that patients with mood disturbances have cerebral hypoperfusion [68–70]. Based on the above studies, being positive for β2GPI-Abs results among patients with NPSLE in cerebral blood flow dysfunction causes mood disorder and stroke occurrence. Nonetheless, there is still a need to study the types of autoantibodies and the specific manifestations of NPSLE.

Mahler M et al. [71], with a large sample size, discovered that anti-Rib-P Ab is a highly specific biomarker for SLE and is frequently associated with NPSLE. One meta-analysis by Shi ZR et al. [72] revealed a strong relationship between anti-Rib-P Ab and NPSLE (OR = 2.72). Another meta-analysis by Ho RC et al. [63] showed a significantly increased prevalence of anti-Rib-P Ab (OR = 2.29) in patients with NPSLE. The results of our study are consistent with those of these previous studies. Our multivariable analysis revealed that anti-Rib-P Ab is associated with a high risk of NPSLE. Abdel-Nasser AM et al. [49] found that a significantly higher proportion of patients with NPSLE are anti-Rib-P Ab positive than other patients, which has a high specificity for detecting depression. Previous research has shown that anti-Rib-P Ab reacts with endothelial cells, thereby affecting the integrity of the BBB [73,74]. Another meta-analysis showed that the C-terminal 22-amino-acid peptide (C22), a key conserved epitope of the anti-Rib-P Ab, is linked to depression (OR = 3.96) and psychosis (OR = 3.99) among patients with NPSLE [75]. An animal study by Wang X et al. [76] revealed that anti-Rib-P Ab can directly cause dysfunction in auditory-evoked potentials in the brain, resulting in neuropsychiatric dysfunction. Another animal study showed that anti-Rib-P Ab acts on the surface of hippocampal neurons, resulting in apoptosis or functional perturbations. Circulating anti-Rib-P Ab can access the hippocampus and impair memory upon disruption of BBB, resulting in cognitive impairment in SLE [77]. Furthermore, a previous *in vitro* study found that anti-Rib-P Ab enters the neuronal cells via the neuronal growth-associated protein, thereby altering neuronal cells. The above studies suggest that anti-Rib-P Ab causes BBB dysfunction by affecting the vascular endothelial cells and the function of neuronal cells as well as the hippocampus. This eventually results in the manifestations of NPSLE, such as depression, mental disorders, and cognitive impairment. To better elucidate the pathological mechanism of anti-Rib-P Ab in NPSLE, it is critical to separately explore its different NP manifestations in future studies with larger sample sizes. Our results are significant as a reference to guide early screening and intervention for NPSLE in children with SLE.

The major strength of this study is that it is a multi-center study focusing on different autoantibodies and clinical manifestations linked to NPSLE in children. However, this work has several limitations. First, several NP manifestations may not be fully reflected due to the small sample size. Secondly, we focused on the laboratory tests and clinical manifestations at the onset of the disease and did not follow up with the patients for long. Thirdly, data on some clinical confounding factors may have gone unnoticed due to the retrospective nature of our study. Therefore, prospective studies are necessary to validate these findings and elucidate the pathological mechanism that underlies NPSLE.

## Conclusion

In conclusion, our study findings confirmed that high SLEDAI-2K score, positive β2GPI-Abs, and anti-Rib-P Ab at the time of SLE diagnosis are predictors of NPSLE. Neuropsychological testing is important in NPSLE, and the pathological mechanism of NPSLE in children warrants further studies.

## Supporting information

S1 Table The raw data. (PDF)

S2 Table Clinical characteristics and neuroimaging/electrophysiological findings in 23 patients with NPSLE. (PDF)
