# Supplementary material for: Predictors of neuropsychiatric manifestations in pediatric patients with lupus
Source: PLoS One. 2025 Jun 10;20(6):e0325915. doi: 10.1371/journal.pone.0325915 (PMC12151350; doi:10.1371/journal.pone.0325915)
Supplement: S1 Table — (PDF) [file pone.0325915.s001.pdf]

Grouping	Time from onset to diagnosis (days)	Age (years)	SLEDAI-2K	ANA	C3	C4	Sex	Anti-dsDNA Ab	Anti-Sm Ab	Anti-CENP Ab	Anti-PCNA Ab	Anti-NU Ab	Anti-HI Ab	Anti-Rib-P Ab	Anti-AMA M2 Ab	Anti-U1RNP Ab	Anti-SSA Ab	Anti-SSB Ab	Anti-Scl70 Ab	Anti-PM Scl Ab	ANCA	LAC	aCL Abs	β2GPI Abs	Hematologic disorder	Renal disorder	Rash	Fever	Pulmonary disorder	Digestive disorder	Arthritis	Cardiac disorder	MAS	Hypothyroidism
1	14	13	23	1000	0.2	0.1	1	1	0	0	0	0	0	0	1	0	0	0	0	0	1	0	1	1	1	1	1	1	0	0	0	0	0	0
1	7	13.4	13	1000	0.2	0	2	0	1	0	0	1	0	1	0	1	1	0	0	0	1	1	0	0	0	0	0	1	1	0	0	0	0	0
1	7	14	21	1000	0.3	0	2	1	0	0	0	1	1	0	1	1	0	0	0	0	1	1	1	1	1	1	0	1	1	0	0	0	0	0
1	9	12	24	3200	1.4	0.1	2	1	0	0	0	1	1	1	0	0	1	1	0	1	0	1	0	0	1	1	1	0	0	1	0	0	0	1
1	165	11	21	1000	0.2	0	2	0	0	0	0	0	0	0	0	0	0	0	0	0	1	0	1	0	1	1	0	0	0	0	0	0	0	1
1	30	7	21	3200	0.4	0.1	2	1	0	0	0	1	0	0	1	0	0	0	0	0	1	1	1	1	1	0	0	1	0	0	1	0	0	0
1	10	11	25	100	0.2	0	2	1	0	0	0	0	0	0	0	0	1	0	0	0	0	0	0	0	1	1	1	1	1	1	0	1	0	1
1	30	12	24	3200	0.2	0	2	1	1	0	0	1	1	1	1	1	0	0	0	0	1	1	1	0	1	1	1	1	1	1	0	0	1	1
1	30	13	26	1000	1	0.1	2	1	1	0	0	1	1	1	0	1	0	0	0	0	1	1	0	1	1	1	1	1	0	1	0	0	0	0
1	150	8	26	3200	0.3	0	2	1	0	0	0	1	1	1	0	0	1	1	0	0	1	1	1	0	1	1	1	1	0	1	0	0	0	1
1	60	13	32	3200	0.4	0.1	2	1	0	0	0	0	1	0	1	0	0	0	0	1	1	0	0	0	1	1	1	1	1	1	0	0	0	1
1	30	10	18	3200	2.3	0.1	2	1	0	0	0	1	1	0	1	0	0	1	0	1	1	1	1	1	1	1	1	1	0	0	1	0	0	1
1	270	11	33	3200	0.3	0.3	2	1	1	0	0	1	1	1	1	1	1	0	0	0	0	0	0	0	1	1	1	1	0	1	1	0	1	0
1	180	7	28	3200	0.3	0	2	1	1	0	0	1	1	1	1	1	0	0	0	0	1	1	0	0	1	0	1	1	0	1	0	0	0	1
1	510	12	17	1000	0.2	0.1	2	1	0	0	0	1	1	1	0	0	0	0	0	0	0	0	1	1	1	0	1	0	0	1	0	0	0	0
1	30	11	42	3200	0.2	0	2	1	0	1	0	1	1	1	1	1	1	1	0	1	1	1	0	0	1	1	0	1	0	1	0	1	0	1
1	25	8	12	320	0.4	0.1	2	0	0	0	0	0	0	0	0	0	0	0	0	0	0	0	0	0	0	0	0	0	0	0	1	0	0	0
1	28	12	18	3200	0.2	0	1	0	1	1	0	0	0	1	0	1	1	0	0	0	0	1	0	1	0	1	0	0	0	1	0	1	0	1
1	12	12	19	3200	0.2	0	2	1	0	0	1	1	1	1	0	1	0	1	0	0	1	0	0	0	1	1	1	1	0	0	0	0	0	0
1	19	12	28	3200	0.2	0	2	1	0	0	0	1	1	1	0	0	0	1	0	0	1	1	0	1	1	1	0	1	1	1	1	0	1	0
1	30	13	22	3200	0.3	0	2	1	1	0	0	1	1	0	1	1	0	1	1	1	1	1	0	0	1	1	1	1	1	0	0	1	0	1
1	90	10	30	3200	0.2	0	2	1	0	0	0	1	1	1	0	0	0	1	0	1	1	0	0	1	1	1	1	1	0	0	1	0	0	1
1	21	10	19	1000	0.3	0.1	2	0	1	0	0	0	0	0	1	1	1	0	0	0	0	1	0	1	1	1	1	1	0	1	0	0	1	1
0	35	14	11	320	0.4	0.1	1	0	1	0	0	0	0	1	0	1	1	0	0	0	1	0	0	0	1	1	0	0	0	0	0	0	0	0
0	38	14	6	100	1	0.2	2	0	1	0	0	0	0	0	0	1	1	0	0	0	0	1	0	0	0	0	1	1	0	0	0	0	0	0
0	36	11	14	320	0.1	0	1	1	0	0	0	1	0	0	0	0	1	0	0	0	1	0	1	0	1	1	0	0	0	0	0	0	0	0
0	49	11.8	14	1000	0.4	0.1	2	1	1	0	0	1	1	1	0	1	1	0	1	0	1	0	0	0	0	1	1	0	0	1	0	0	0	0
0	37	11	16	1000	0.1	0.1	2	1	0	0	0	1	1	0	1	0	0	0	0	0	0	1	0	1	1	1	1	1	1	0	0	0	0	0
0	182	11.8	4	320	0.6	0.1	2	0	1	0	0	0	0	1	0	0	0	0	0	0	0	1	0	0	0	0	1	0	0	0	0	0	0	0
0	10	12.7	19	1000	0.1	0.1	2	0	0	0	0	1	0	0	0	0	0	0	0	0	1	0	0	0	0	1	0	1	1	0	0	0	0	0
0	30	15	7	320	0.9	0.1	2	0	0	0	0	0	0	0	0	0	0	0	0	0	0	0	0	1	1	0	0	0	0	0	0	0	0	0
0	10	10.5	15	320	0.7	0.1	2	0	1	0	0	0	0	1	0	1	0	0	0	0	1	1	1	1	0	0	1	1	0	0	0	0	0	0
0	365	13.5	8	320	0.2	0	1	0	1	0	0	0	0	0	0	0	0	0	0	0	1	0	0	0	0	0	1	0	0	0	0	0	0	0
0	7	11.4	13	1000	0.2	0.1	1	1	0	0	0	1	1	0	1	0	0	0	0	0	1	0	1	0	1	1	0	0	0	0	0	0	0	0
0	120	10.6	2	320	0.4	0.1	2	0	0	0	0	1	1	1	0	1	0	0	0	0	1	0	1	1	0	0	1	0	0	0	0	0	0	0
0	30	12.6	13	1000	0.6	0.1	2	1	0	0	0	1	1	0	0	0	1	0	0	0	1	1	1	0	0	1	0	1	0	0	0	0	0	0
0	7	14	15	320	0.7	0.1	2	0	0	0	0	0	0	0	0	0	0	0	0	0	0	0	0	0	1	1	0	1	1	1	0	0	0	0
0	182	7	14	320	0.4	0	2	0	0	0	0	0	1	1	0	0	0	0	0	0	1	1	1	0	0	1	1	0	0	0	1	0	0	0
0	90	13.3	10	100	0.4	0.1	2	0	1	0	0	0	0	0	0	1	0	0	0	0	0	0	0	0	1	1	1	0	0	0	0	0	0	0
0	60	13.3	14	1000	0.2	0	2	1	1	0	0	1	1	0	0	1	0	0	0	0	1	0	0	0	1	1	1	0	0	0	0	0	0	0
0	7	13.4	11	1000	1	0.2	2	0	0	0	0	0	0	0	0	0	1	1	0	0	0	1	0	0	0	1	1	1	1	0	0	0	0	0
0	15	10.3	4	1000	0.9	0.1	1	0	0	1	0	0	0	0	0	1	0	0	0	0	0	1	0	0	1	0	0	1	1	0	0	0	0	0
0	567	8	3	1000	0.8	0.1	1	0	0	0	0	0	1	0	0	0	0	0	0	0	0	1	0	1	1	0	1	0	0	1	0	0	0	0
0	8	8.8	9	1000	1	0.2	2	0	0	0	0	0	0	0	0	0	0	0	0	0	0	0	0	1	1	1	0	0	0	1	0	0	0	0
0	7	4.8	3	1000	0.6	0.1	2	0	0	0	0	0	0	0	0	0	1	0	0	0	0	0	0	0	1	0	0	0	0	0	0	0	0	0
0	14	10.7	16	1000	0.2	0	2	0	0	0	0	1	1	0	0	0	1	1	0	0	1	0	0	0	1	1	0	1	0	0	0	0	0	0
0	7	10.8	16	1000	0.8	0.2	2	0	0	0	0	1	1	0	0	0	0	0	0	0	1	1	1	0	1	1	1	1	0	0	0	0	0	0
0	8	7	8	1000	0.1	0	1	1	1	0	0	1	1	0	0	1	1	0	0	0	1	0	0	0	1	0	1	1	0	1	0	0	0	0
0	60	12	4	1000	0.5	0.1	2	1	1	0	0	1	1	0	0	1	0	0	0	0	0	1	0	0	0	0	0	0	0	0	0	0	0	0
0	10	11	15	1000	0.4	0.1	2	0	0	0	0	0	0	0	1	0	1	0	0	0	0	0	0	0	1	1	0	0	0	0	0	0	0	0
0	15	10	7	1000	0.4	0.1	2	0	0	0	0	1	1	1	0	1	0	0	0	0	1	1	0	0	1	0	1	0	0	0	0	0	0	0
0	26	10	12	1000	0.5	0	2	1	0	0	0	1	1	1	0	0	0	0	0	0	0	0	0	0	0	1	0	0	0	0	0	0	0	0
0	28	4.6	2	100	2	0.1	2	0	1	0	0	0	0	0	0	1	0	0	0	0	0	1	0	0	0	0	1	1	0	0	0	0	0	0
0	30	12	2	1000	0.9	0.2	2	0	0	0	0	0	1	0	0	0	0	0	0	0	0	0	0	0	1	0	0	1	0	0	0	0	0	0
0	25	13.5	9	1000	0.6	0.2	1	0	0	0	0	1	1	0	0	0	1	0	0	0	0	0	0	0	1	1	0	1	0	0	0	0	0	0
0	240	11.6	3	320	0.6	0.2	2	0	0	0	0	1	0	0	0	0	0	0	1	0	0	0	1	1	1	0	0	0	0	0	0	0	0	0
0	7	11	4	100	0.8	0.1	2	0	0	0	0	0	0	0	0	0	1	1	0	0	0	0	0	0	1	0	0	1	0	0	0	0	0	0
0	9	9	14	1000	0.2	0	2	1	0	0	0	1	1	0	0	0	0	0	0	0	1	0	1	0	1	1	0	0	0	0	0	0	0	0
0	150	9	12	320	0.6	0.1	1	0	1	0	0	0	0	1	0	1	0	0	0	0	0	1	0	0	1	0	1	1	0	0	1	0	0	0
0	14	7	13	100	0.2	0	2	1	1	0	0	1	1	0	1	1	0	0	0	0	0	0	0	0	1	1	1	1	0	0	0	0	0	0
0	14	12	15	1000	0.2	0	2	1	0	0	0	1	1	0	0	0	1	0	0	0	1	0	0	0	0	1	1	1	0	0	0	0	0	0
0	15	11	21	320	0.3	0	2	1	0	0	0	1	1	0	0	0	0	0	0	0	0	1	1	1	1	1	1	0	1	0	0	1	0	1
0	150	9	19	3200	0.5	0	2	0	1	0	0	0	0	1	0	1	1	0	0	0	0	0	0	0	1	1	1	1	1	1	0	0	0	1
0	15	9	21	100	0.4	0.1	1	0	0	0	0	0	0	0	1	0	0	0	0	0	0	0	0	0	1	1	1	1	0	0	0	0	0	1
0	60	11	16	1000	1.1	0.1	1	1	0	0	0	0	0	0	0	0	1	0	0	0	0	0	0	0	1	1	1	0	0	0	0	0	0	0
0	120	12	29	3200	0.4	0.1	2	1	0	0	0	1	1	1	1	0	0	0	0	1	1	0	0	0	1	1	1	1	1	1	0	1	0	1
0	11	8	16	100	1.1	0.2	2	1	0	0	0	0	0	0	0	0	1	0	0	0	0	0	0	0	0	1	0	0	0	1	0	0	0	1
0	32	11	16	3200	0.2	0	2	1	0	0	0	1	1	0	0	0	1	1	0	1	1	0	1	0	1	1	0	0	0	0	0	0	0	1
0	30	11	29	3200	0.3	0.1	2	1	0	0	0	1	1	0	1	0	0	1	0	1	1	0	0	0	1	1	1	1	1	0	1	0	0	1
0	14	11	20	1000	1.2	0.4	2	1	0	0	0	0	0	0	0	0	1	0	0	0	0	0	0	1	1	1	0	1	0	0	0	0	0	1
0	19	9	23	1000	0.5	0.1	2	1	0	0	0	0	0	0	0	0	1	0	0	0	1	0	0	0	1	1	0	0	1	0	0	1	0	1
0	30	11	31	3200	0.2	0	2	1	0	0	0	1	1	0	0	0	0	0	0	0	1	1	0	0	1	1	0	0	0	1	0	0	0	1
0	30	7	20	3200	0.2	0	2	1	0	0	0	1	0	0	1	1	1	0	0	0	0	0	0	0	1	1	1	0	1	1	0	0	0	1
0	30	14	22	1000	0.5	0.1	2	0	0	0	0	0	0	0	0	1	1	0	0	0	0	0	1	0	1	1	0	0	0	1	0	1	0	0
0	90	9	24	3200	0.3	0	2	1	0	0	0	1	1	1	0	0	0	0	0	0	0	0	0	0	1	1	1	1	0	0	0	1	0	1
0	90	14	26	1000	0.2	0	2	1	0	0	0	1	0	1	0	0	0	0	0	0	1	0	0	0	0	1	1	0	0	1	0	0	0	0
0	120	11	23	3200	0.4	0.1	2	1	1	0	0	1	1	0	1	1	1	1	0	1	1	0	0	0	0	1	1	1	0	0	1	0	0	1
0	4	12	17	3200	0.2	0	2	1	0	0	0	1	0	0	0	0	1	1	0	0	0	0	0	0	1	1	0	0	0	0	0	0	0	1
0	30	6	18	3200	0.5	0	2	1	0	0	0	1	1	1	1	1	0	0	0	0	1	0	0	1	1	0	1	1	0	0	0	0	0	1
0	60	12	20	1000	0.1	0	2	1	0	0	0	1	1	0	1	0	0	0	0	0	1	0	1	0	1	1	0	1	0	0	0	0	0	1
0	14	13	18	320	0.5	0.1	2	1	0	0	0	0	0	0	0	0	0	0	0	0	0	0	0	0	0	1	1	0	1	0	0	0	0	0
0	9	10	20	1000	0.4	0.1	1	1	0	0	0	1	1	0	0	0	0	0	0	0	0	1	1	0	1	1	0	0	0	0	0	0	0	1
0	11	7	24	3200	0.3	0	2	1	0	0	0	1	1	0	1	0	0	0	0	0	0	0	0	0	0	1	1	0	0	0	1	0	0	1
0	30	12	8	3200	0.5	0	2	1	1	0	0	0	0	0	0	1	1	1	0	0	0	0	0	0	0	1	0	0	0	0	0	0	0	0
0	22	13	7	1000	0.5	0	2	1	0	0	0	0	0	1	0	0	0	0	0	0	1	0	0	0	1	0	1	0	0	0	0	0	0	0
0	20	13	22	1000	0.2	0.1	2	1	0	0	0	1	1	0	0	0	0	0	0	0	1	0	0	1	1	1	1	0	0	0	0	0	0	1
0	30	11	26	3200	0.7	0.1	2	1	1	0	0	1	0	0	1	1	1	1	0	0	0	0	0	0	0	1	1	1	0	0	0	1	0	0
0	60	4	14	3200	0.4	0.2	1	0	0	0	0	0	0	0	0	0	0	0	0	0	0	0	0	0	0	1	0	0	0	0	0	0	0	1
0	9	5	14	320	0.2	0	2	0	0	0	0	1	0	0	0	0	1	1	0	0	0	0	0	0	1	0	0	0	0	0	0	0	0	0
0	90	11	26	3200	0.2	0	2	1	1	0	0	1	1	0	1	1	0	0	1	1	0	1	0	0	1	1	1	1	0	1	1	0	0	1
0	30	10	25	3200	0.3	0	2	1	0	0	0	1	1	0	0	1	1	0	0	0	1	0	0	0	1	1	1	1	0	1	1	0	0	0
0	21	13	12	3200	1.3	0.1	2	0	1	0	0	0	0	0	0	1	1	0	0	0	0	1	0	1	0	1	0	0	0	0	1	0	0	0
0	150	13	12	3200	0.2	0	2	1	1	0	1	1	1	0	1	1	0	0	0	0	1	0	0	0	0	1	0	1	0	1	0	1	0	0
0	8	11	5	1000	0.9	0.2	2	1	0	0	0	0	0	0	1	0	0	0	0	0	0	0	0	0	1	0	0	0	0	0	0	0	0	0
0	21	12	15	3200	0.3	0	2	1	1	0	0	1	1	0	1	1	0	1	0	1	0	0	0	0	1	1	1	0	0	1	1	1	0	0
0	15	7	18	1000	1.3	0.5	2	0	0	0	0	0	0	0	0	1	1	1	0	0	0	0	0	1	1	1	1	1	0	1	0	0	0	0
0	9	10	22	3200	0.7	0.1	2	0	1	0	0	0	0	0	0	1	1	0	0	0	0	0	0	0	1	0	1	1	1	1	0	1	0	1
0	18	13	19	1000	0.2	0	2	1	1	0	0	1	1	0	0	1	0	1	0	1	0	0	0	0	1	1	0	1	0	0	0	0	0	0
0	30	10	9	3200	0.4	0	2	1	1	0	0	1	0	0	0	1	0	0	0	0	0	0	0	0	0	1	1	1	0	0	0	0	0	0
0	365	11	5	1000	0.6	0.1	2	1	0	0	0	1	0	1	1	0	1	0	0	0	1	0	0	0	0	1	1	0	0	0	0	0	0	0
0	18	10	19	320	0.3	0	1	1	1	0	0	1	1	1	0	1	0	0	0	0	0	0	0	1	1	1	1	0	0	0	0	0	0	1

Note:
For groups: 1 indicated the NPSLE group, 0 indicated the SLE without NPSLE group.
For gender: 1 indicated male, 2 indicated female.
For antibodis: 1 indicated positive, 0 indicated negative.
For organ involvement: 1 indicated involvement, 0 indicated no involvement.
