# Supplementary material for: Predictors of neuropsychiatric manifestations in pediatric patients with lupus
Source: PLoS One. 2025 Jun 10;20(6):e0325915. doi: 10.1371/journal.pone.0325915 (PMC12151350; doi:10.1371/journal.pone.0325915)
Supplement: S2 Table — (PDF) [file pone.0325915.s002.pdf]

Supplementary Table 2. Clinical characteristics and neuroimaging/electrophysiological findings in 23 patients with NPSLE

No.	Clinical Manifestations (Neuropsychiatric syndromes)	Brain MRI Findings	EEG Findings
1	Headache (Headache)	Atrophy	NL
2	BP lability, Hyperhidrosis (Autonomic disorder)	NL	Moderate encephalopathic changes
3	Status epilepticus (Status epilepticus)	Abnormal signal	-
4	Orthostatic hypotension, Dizziness (Autonomic disorder)	Abnormal signal	NL
5	Hyperhidrosis, Dizziness (Autonomic disorder)	Abnormal signal	NL
6	Headache, Hemiparesis (Cerebrovascular Disease)	Abnormal signal	-
7	Psychomotor retardation, Depression (Mood disorder)	Abnormal signal + Atrophy	NL
8	Psychomotor retardation, impaired calculation ability with cognitive dysfunction, and bilateral upper limb tremors (Cognitive dysfunction) (Movement disorder)	Abnormal signal	NL
9	Headache (Headache)	Atrophy	-
10	Hypotonia (Movement disorder)	Atrophy	Diffuse background slowing
11	Headache (Headache)	Abnormal signal + Atrophy	NL
12	Psychomotor retardation, Depression (Mood disorder)	Abnormal signal	-
13	Consciousness disturbance; disorganized speech (Acute confusional state)	Abnormal signal	-
14	Headache, Sensory deficit (Cerebrovascular Disease)	Abnormal signal + Atrophy	NL
15	Headache; Hypotension (Autonomic disorder)	Abnormal signal	NL
16	Anxiety, Sensory deficit (Cerebrovascular Disease)	Abnormal signal	-
17	Headache (Headache)	Abnormal signal	NL
18	Impaired calculation ability; Memory impairment (Cognitive dysfunction)	Abnormal signal	NL
19	Depression, Anhidrosis (Autonomic disorder)	Abnormal signal + Atrophy	NL
20	Headache, Hemiparesis (Cerebrovascular Disease)	Abnormal signal	Diffuse background slowing
21	Psychomotor retardation, Depression (Mood disorder)	Abnormal signal	-
22	Anxiety (Mood disorder)	Abnormal signal + Atrophy	NL
23	Facial tics; Limb tremors; Constipation (Movement disorder) (Autonomic disorder)	Abnormal signal	Diffuse background slowing and frequent polymorphic delta slow waves in the prefrontal area during wakeup period

Abbreviations: BP: blood pressure; NL: normal; -: test not performed.
MRI Findings: "Abnormal signal" = Brain parenchyma abnormal signal; "Atrophy" = Brain atrophy.
